# A method for differentiating human induced pluripotent stem cells toward functional cardiomyocytes in 96-well microplates

**DOI:** 10.1038/s41598-020-73656-2

**Published:** 2020-10-28

**Authors:** Novin Balafkan, Sepideh Mostafavi, Manja Schubert, Richard Siller, Kristina Xiao Liang, Gareth Sullivan, Laurence A. Bindoff

**Affiliations:** 1 Department of Clinical Medicine (K1), University of Bergen, Bergen, Norway; 2 Department of Neurology, Haukeland University Hospital, 5021 Bergen, Norway; 3 Department of Molecular Medicine, Institute of Basic Medical Sciences, University of Oslo, Oslo, Norway; 4 Norwegian Center for Stem Cell Research, Oslo University Hospital, Domus Medica, Oslo, Norway; 5 Institute of Immunology, Oslo University Hospital-Rikshospitalet, P.O. Box 4950, 0424 Nydalen, Oslo, Norway; 6 Hybrid Technology Hub - Centre of Excellence, Institute of Basic Medical Sciences, University of Oslo, Blindern, Oslo, Norway; 7 Neuro-SysMed, Department of Neurology, Haukeland University Hospital, Bergen, Norway

**Keywords:** Stem cells, Heart stem cells, Pluripotent stem cells, Stem-cell differentiation

## Abstract

The capacity of pluripotent stem cells both for self-renewal and to differentiate into any cell type have made them a powerful tool for studying human disease. Protocols for efficient differentiation towards cardiomyocytes using defined, serum-free culture medium combined with small molecules have been developed, but thus far, limited to larger formats. We adapted protocols for differentiating human pluripotent stem cells to functional human cardiomyocytes in a 96-well microplate format. The resulting cardiomyocytes expressed cardiac specific markers at the transcriptional and protein levels and had the electrophysiological properties that confirmed the presence of functional cardiomyocytes. We suggest that this protocol provides an incremental improvement and one that reduces the impact of heterogeneity by increasing inter-experimental replicates. We believe that this technique will improve the applicability of these cells for use in developmental biology and mechanistic studies of disease.

## Introduction

Human pluripotent stem cells, hPSCs, including human embryonic cells, ESCs and human induced pluripotent stem cells, iPSCs, are increasingly used in both drug discovery and to study disease and tissue development. An important feature of human pluripotent stem cells (hPSCs) is their ability to differentiate into virtually any cell type including cardiomyocytes^1–3^. Previous studies have shown that stem cell-derived cardiomyocytes share characteristics and functional properties of primary human heart tissue^4–6^. Monolayer directed differentiation, together with a combination of small molecules and well-defined culture media have enhanced the generation of cardiomyocytes at relative high purity (> 80%) without the need for additional growth factors^7–9^. Variation in differentiation efficiency and low reproducibility have nevertheless remained problematical and these have been variously ascribed to initial seeding densities and confluency and the effect of batch variability of compounds used in the differentiation process^7,10–12^. Furthermore, inter- and intra-clonal variation in both hESC and hiPSC lines, as demonstrated by differences in gene and protein expression, DNA methylation, and differentiation potential, have a major impact on the efficiency of differentiation^8,13–16^. To overcome such problems, and provide an enabling platform for studying etiology of the disease in early developmental stages, we investigated cardiomyocyte differentiation using defined media and small molecules in a 96-well plate format. Compared with larger plate formats, the 96-well plate format allows an increased number of replicates, giving a greater potential for reproducibility, while still being cost-effective.

## Results

### Optimizing cardiomyocyte differentiation in 96 well format

**Figure 1:**
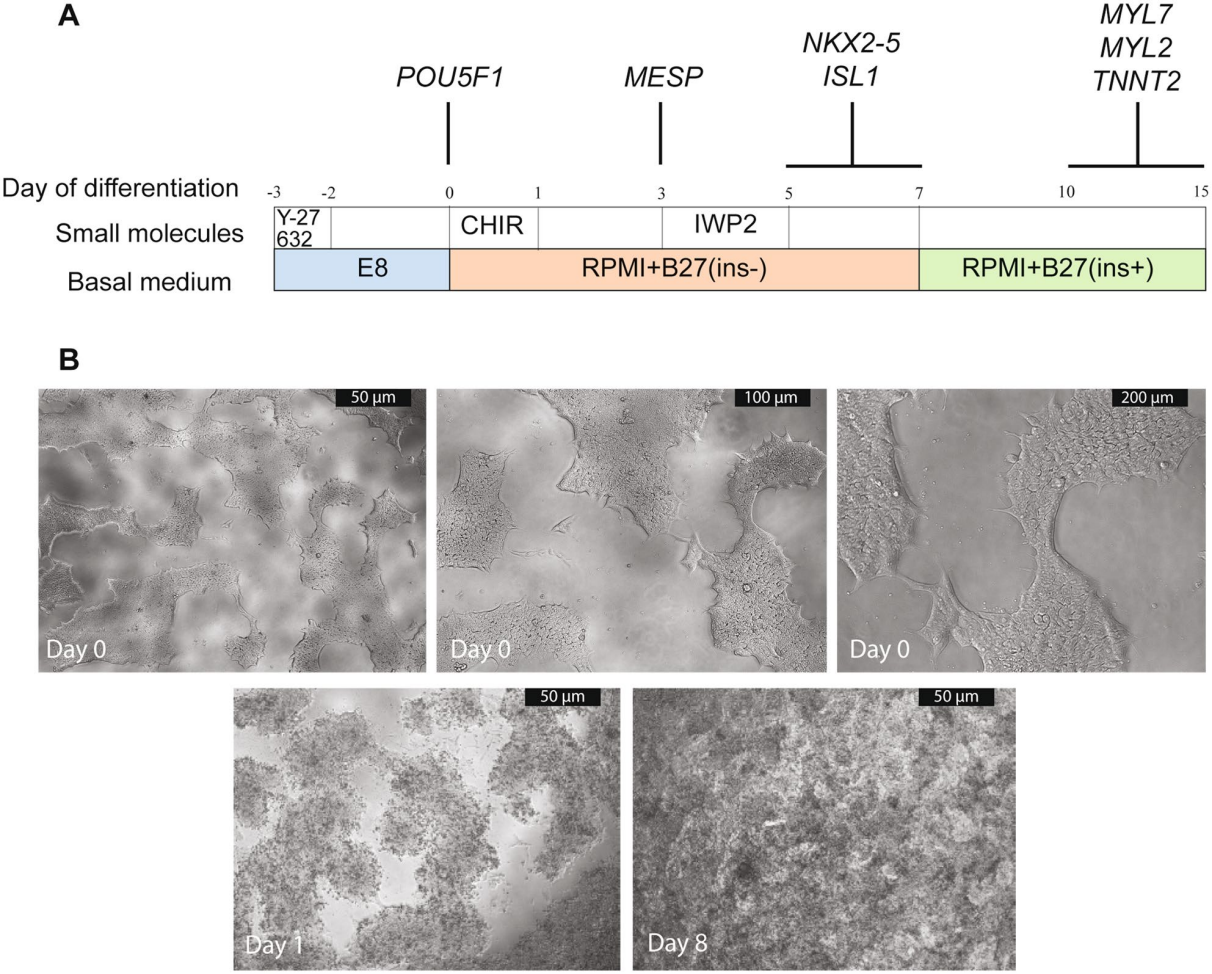
An overview of the protocol. (**A**) Schematic of representation of differentiation protocol from hPSCs to differentiated cardiomyocytes. The different media and small molecules used at each stage are shown together with the markers that define the stage of differentiation. (**B**) Phenotypic overview of H1 culture at different differentiation stages. Differentiation was started when the confluency of the culture reached 60–70% (Day 0). The quality of the hPSC colonies was checked visually (i.e., homogeneous appearing colonies with clear borders and the absence of obvious differentiating zones). Cells were treated with 6 µM (hiPSCs) or 8 µM (hESCs) CHIR99021 (CHIR) on day 1 for 24 h followed by treatment with IWP2 for 48 h on day 3. By day 8, beating islands of cardiomyocytes covered most of the space in each well. Pictures were taken using inverted light microscopy (Leica DML 1000). Scale bars, 50 μm. The quality of the hPSC colonies was investigated with higher magnifications, 100 and 200 μm.

As differentiation yield is affected by the quality of hESCs/hiPSCs, the matrix, media, small molecules, cell density and cell confluency^8,12,17,18^, we evaluated these factors in a 96-well format. We used two hESC lines (H1 and H9) and two hiPSC lines: Detroit 551-A and AG05836B-15^1,19–21^. In order to account for potential differences in the reprogramming methods employed, we used a retroviral reprogrammed line (Detroit 551-A) and an integration free Sendai virus reprogramed line (AG05836B-15). Further, we changed propagation matrix and media to Geltrex and Essential 8 Medium (E8) respectively. Figure 1 shows the time line of differentiation, the media and small molecules used, some of the developmental markers studied and colony morphology.

**Figure 2:**
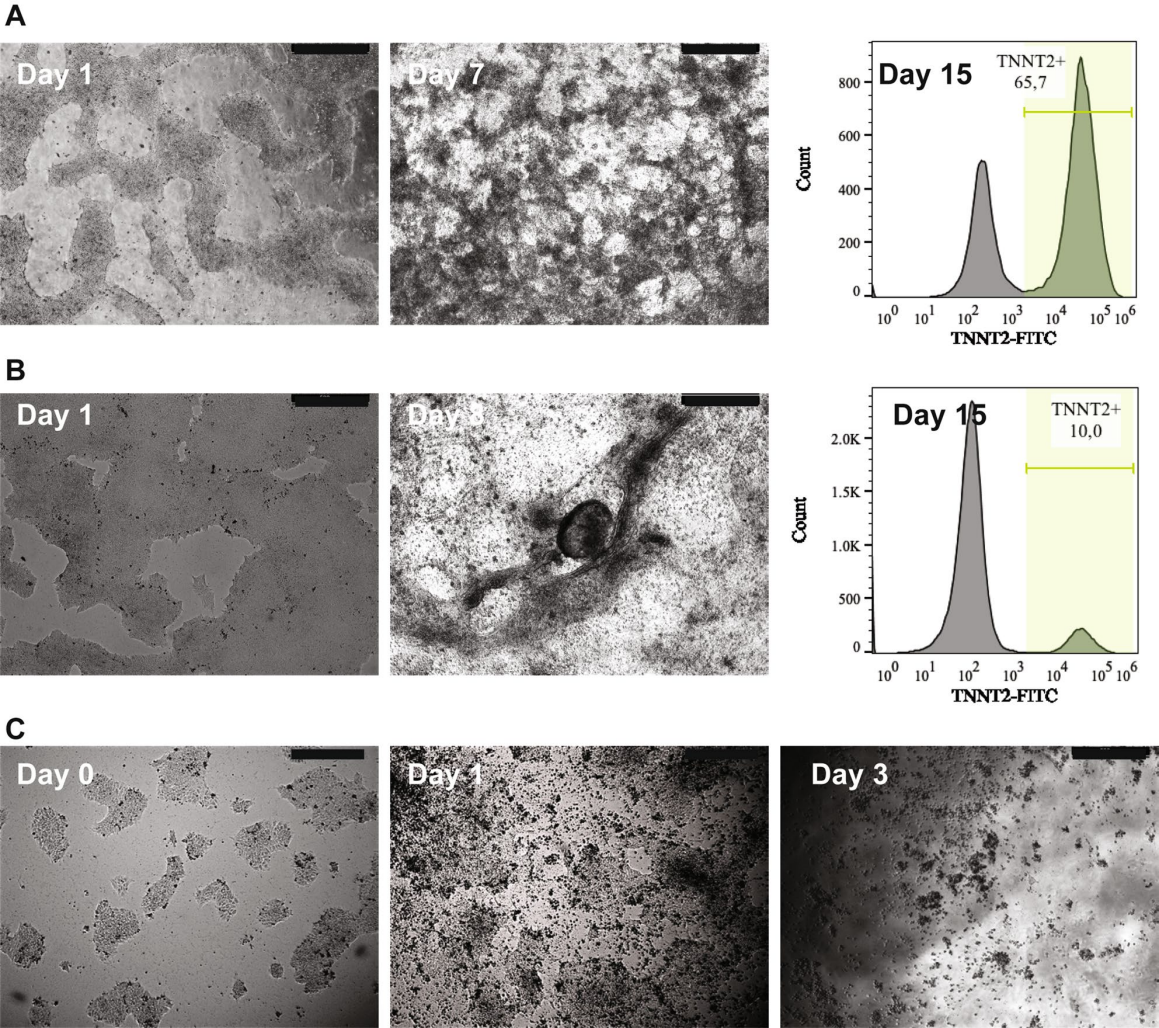
The impact of confluency on differentiation efficiency. The figure shows the morphology of Detroit551-A cell culture with different confluency prior to differentiation initiation and monitoring them at different stages of differentiation process. Finally estimation of TNNT2 + cells in the culture using flow cytometry. (**A**) Cells cultured to a confluency of 60–70% prior to differentiation initiation. Beating cardiomyocytes appeared on day 7 of differentiation and staining the cells for TNNT2 marker showed a high level of TNNT2 + cells on day 15. (**B**) This panel shows Detroit 551-A at a starting confluency of 80–90% on day 1 and the presence of few beating clumps on day 8 after initiation of differentiation. Flow cytometry revealed low level of TNNT2 + cells in the culture on day 15. (**C**) This panel shows morphology of Detroit 551-A cells, cultured to a confluency of 30–40% prior to differentiation. This initial confluency gave rise to no cardiomyocytes. Cells started to die just after 24 h treatment with CHIR99021 (day-1) and most were dead after 3 days. All Pictures were taken using inverted light microscope (Leica DML 1000). Scale bars, 500 μm. Cardiomyocyte population were estimated using flowcytometer Sony cell sorter SH800 (Sony Biotechnology Inc.) and data were analyzed and visualized using FlowJo 10.5.0 (FlowJo LLC, OR, USA) software (www.flowjo.com).

We investigated confluency by initiating differentiation at 30–40%, 60–70% and 80–90% confluency and found that 60–70% confluency gave rise to a culture with the highest percentage of functional cardiomyocytes on day 15 (D15) of differentiation (Fig. 2). Then, we estimated the cell density needed to cover 60–70% of the well area within a minimum of 2 days after cell seeding. Seeding involves resolving hPSC colonies into single cell suspension and/or small clumps containing two to six cells using E8 medium supplemented by Y27632. Cells were incubated at 37 °C overnight in this combination and after 24 h the medium changed to fresh E8 medium. Thus, estimating seeding density required a minimum 2 days in culture to provide enough time for the cells to recover and reach the desired confluency. We identified a density of 2.4 × 10^4^ cells/cm^2^ for both hESCs and hiPSCs as optimal for generating areas of beating cardiomyocytes by day 7 and spontaneous contractions of larger areas by day 9 to 10 (see Supplementary Fig. S1 and Video S1). The lines used in these experiments were 60–70% confluent within 2–3 days, however, time to optimal confluency may vary for other cell lines. Further, we also noted that any deviation in confluency without reciprocal concentration adjustment of the small molecule CHIR99021 resulted in either increased cell death or low differentiation efficiency. We found that 8 μM was optimal for hESCs while for hiPSCs, 6 μM CHIR99021 resulted in high levels of TNNT2^+^ cells on D15.

Human ESCs/hiPSCs were propagated on Geltrex under feeder free conditions in Essential 8 Medium (E8) at 2.4 × 10^4^ cells/cm. Within 3 days, they reached a confluency of 60 to 70%, the ideal point to induce differentiation by applying the GSK3 inhibitor CHIR99021, in concentration-cell-dependent manner. The inhibitor of WNT production-2, IWP2, was added 72 h post differentiation induction for 48 h. Fresh medium was provided (RPMI/B-27 without insulin) on day 5, and from day 7 cells were fed with fresh RPMI/B-27 (with insulin) every two other days (Fig. 1). We observed areas of beating cardiomyocytes by day 7 and by day 9—10, spontaneous contractions could be seen in large areas of the well (see Supplementary Video S1). Contractile cardiomyocytes were collected from day 10—15 for further analysis.

### Characterizing cardiomyocytes derived from human pluripotent stem cells

#### Assessment of developmental markers throughout differentiation

**Figure 3:**
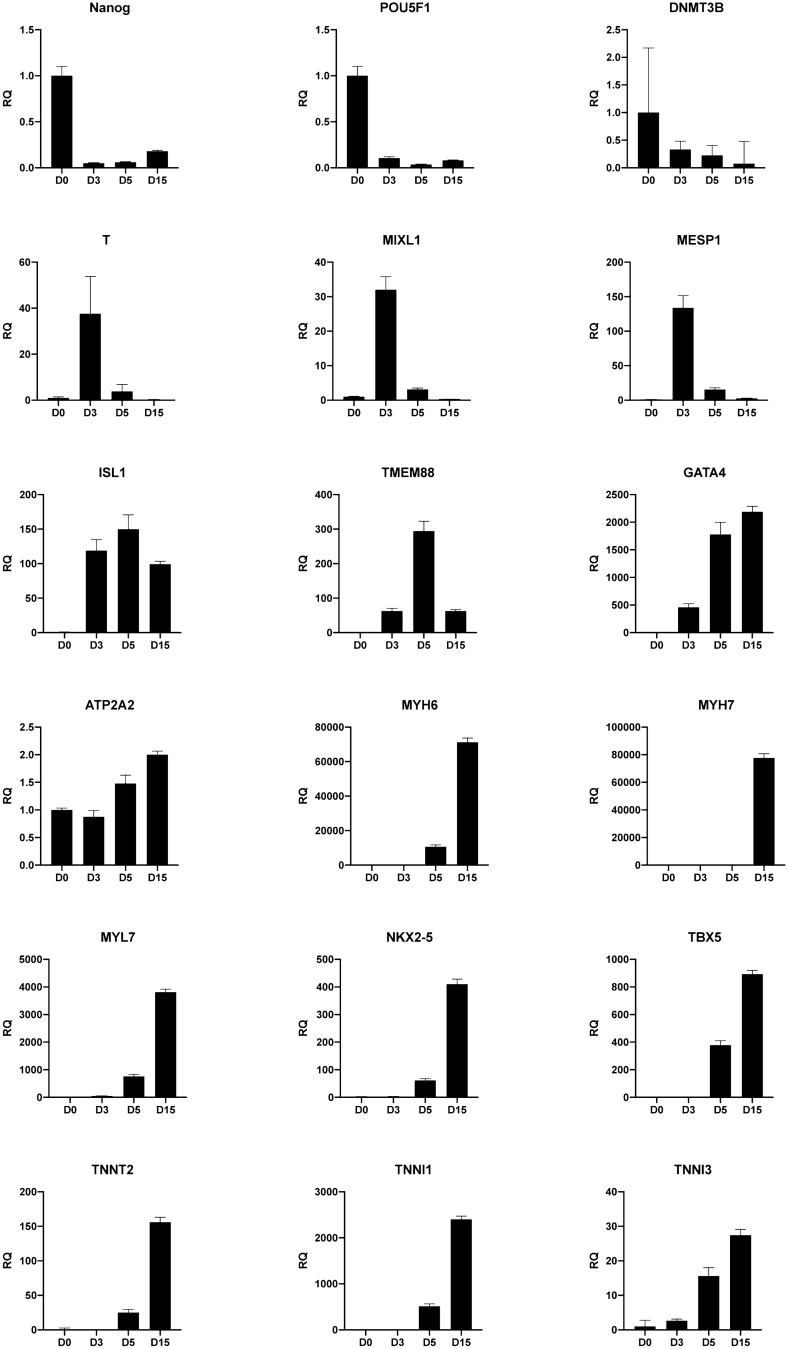
Characterization of H1-derived cardiomyocytes by gene expression. Relative quantification of mRNA expression profiles of 18 markers, representing the different stages of differentiation from hPSC to the cardiac lineage. Data are collected from the differentiation of hESC-H1 toward cardiomyocytes and presented as the mean of three independent differentiation runs and error bars are the standard deviation of the mean. Changes in expression level are related to the pluripotent stage, D0.

Differentiation from hPSC toward the cardiac lineage involves formation of a primitive-streak-like population, from which all endodermal and mesodermal lineages develop including cardiovascular progenies i.e. cardiomyocytes, endothelial cells and smooth muscle cells. To ensure that differentiation followed the correct developmental route, we monitored the expression levels of key markers at time points corresponding to stage-specific transitions including—pluripotency state (D0), germ layer specification (D3), progenitor state (D5) and committed cardiomyocytes (D15) in the ES line H1 (Fig. 3) and we confirmed this in the Detroit IPSC line (Fig. S2). As expected, treatment of hPSC with CHIR99021 resulted in decreased expression of pluripotency genes *NANOG* and *POU5F1*, and the appearance of early mesodermal markers such as *MESP1, MIXL1* and *T* at the highest level on D3 of differentiation (Fig. 3, Supplementary Figs. S2 and S3A). Further differentiation of H1 and Detroit 551-A to cardiac progenitor was defined by the increased expression of *ISL1* and *TEMEM88* on D5*,* and to committed cardiomyocyte defined by the specific markers such as *TBX5*, *TNNT2, MYH6* and *MYL7* (Fig. 3, Supplementary Figs. S2 and S3A). The gene expression data revealed the gradual increase of *GATA4* and *ATP2A2* expression while cells were navigating from germ layer specification (D3) towards the committed cardiomyocytes (D15), and expression of cardiac specific markers *TNNT2* and *MYL7* coincided with maturation and contractile activity (Fig. 3, Supplementary Figs. S2 and S3A). These studies also showed the presence of *MYL2*, a marker for ventricular myocytes, from day 12 (Supplementary Fig. S3A).

To ensure that we were seeing appropriated maturation, we investigated the expression of mature cardiomyocyte markers such as *HOPX* and *MYH7*^*6*^ at a later stage of differentiation, D30. We showed that HOPX and MYH7 increased at D30 of differentiation and MYH6 fell (Supplementary Fig. S4).

**Figure 4:**
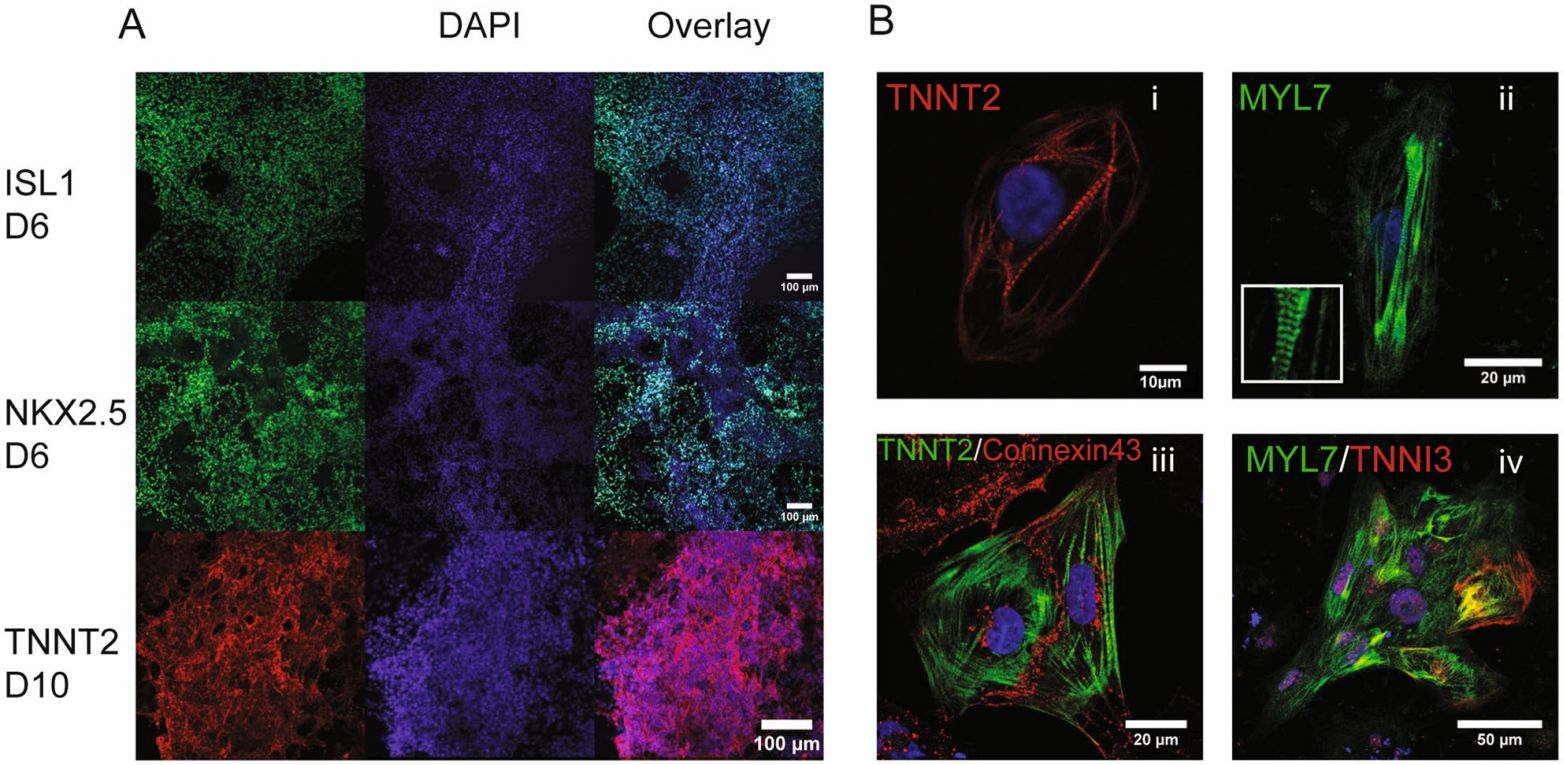
Cardiomyocyte characterization. Cardiomyocyte culture consists of several layers of cells, which makes it difficult to stain the cells in single well of 96 well plate. Cardiomyocyte characterization using immunocytochemistry was performed by transferring cells one well of 96 well plate into a single well of 12 well plate. (**A**) Detroit 551-A cells were differentiated in a 96 well format and transferred at day 5–12-well plates and cultured for a further 1–5 days. They were then stained for markers of cardiomyocyte differentiation (i.e. differentiation time points 6 and 10 days). Scale bar, 100 µm. (**B**) hiPSCs differentiated to CM and transferred on day 8 from 96-well plate to cover slips in a 12-well plate. (i) Detroit 551-A stained with TNNT2; (ii) AG05836B-15 stained with MYL7 showing the typical cardiac sarcomere organization; (iii) hESC-H1 co-stained for conexin43 in TNNT2 positive cells showing the formation of both organized sarcomeres and gap junctions; (iv) hESC-H9 cells are co-stained for TNNI3 and MYL7. Images collected between day 10–12 using Leica TCS SP5 confocal microscope.

We confirmed our gene expression findings using immunocytochemistry (Fig. 4 and Supplementary Fig. S3). We assessed the hPSCs for pluripotency and found expression of POU5F1 and no evidence of the cardiomyocyte marker TNNT2 in hPSC (day 0) prior to initiation of the differentiation. By day 7 of the differentiation process, we observed a marked reduction in the expression of POU5F1 and the appearance of the cardiomyocyte marker TNNT2 (Supplementary Fig. S3B). Next, we assessed cells committed to the cardiac lineage by evaluating the expression of ISL1 and NKX2-5 that define the cardiac progenitor stage. We found ISL1 and NKX2-5 positive cells by day 6 of the differentation (Fig. 4A) and TNNT2 was robustly detected by day 10 (Fig. 4A). Staining with an antibody against myosin light chain 7 (*MYL7*), a specific marker for atrial myocytes, revealed the presence of cardiac sarcomeres in the cells within D10-12 of differentiation (Fig. 4B-ii). Furthermore, we found the expression of connexin 43 (*GJA1*), a marker for the gap junctions in TNNT2^+^ cells on D10-12 of differentiation (Fig. 4B-iii). Co-staining of MYL7 and troponin I3 (*TNNI3*) that is specefic to the cardiac muscle cells (Fig. 4B-iv) confirmed the presence of committed cardiomyocytes in the culture on D10-12 of differentiation.

#### Quantification of cell composition using flow cytometric analysis

**Figure 5:**
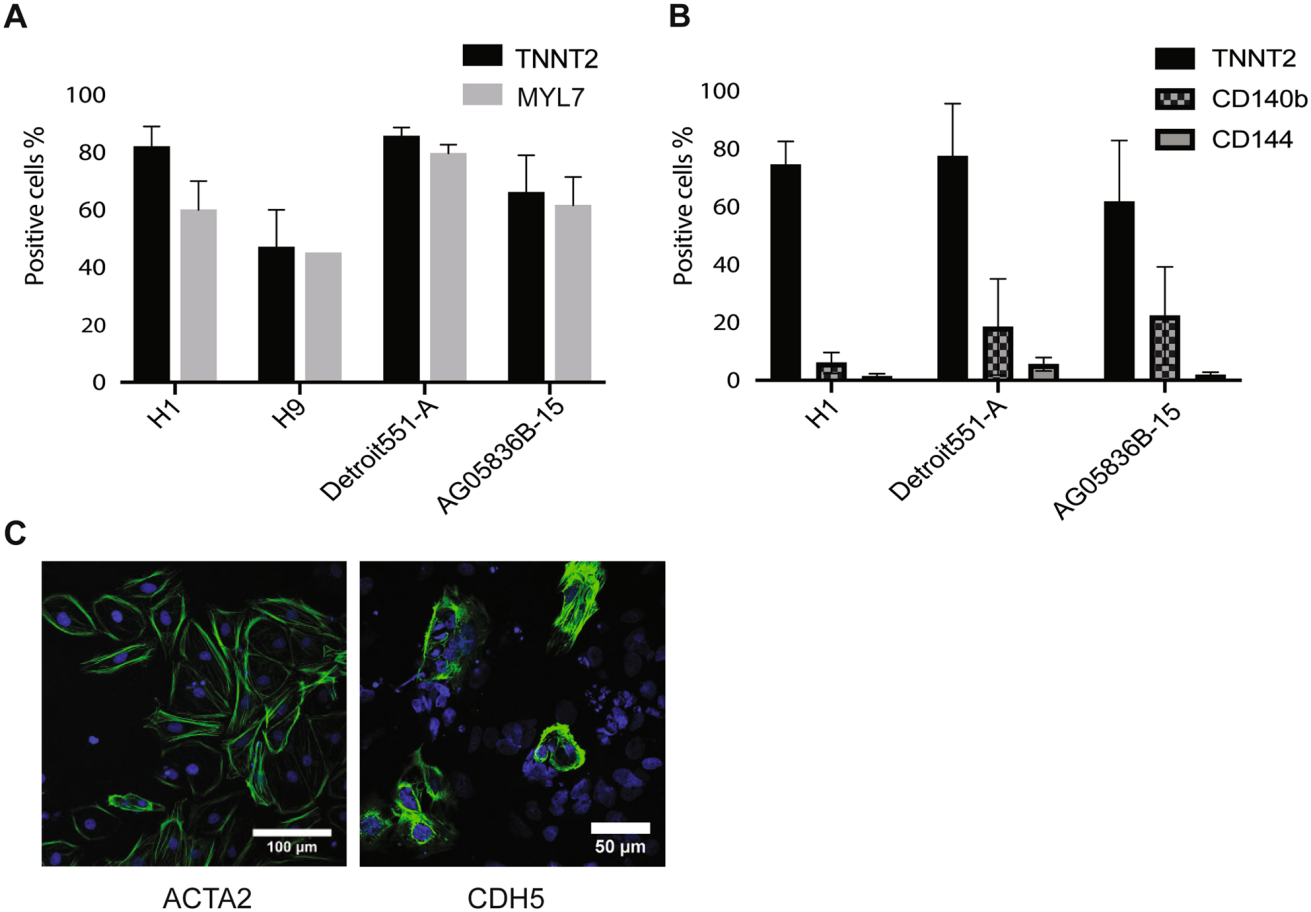
Verification of cardiomyocytes differentiation using flow cytometric analysis. (**A**) Results presented as the percentage of TNNT2 and MYL7 positive cells in different hiPSC lines (Detroit 551-A and AG05836B-15) and hESC lines (H1 and H9). Detroit 551-A (85%) and H1 (82%) showed higher level of TNNT2 positive cells compared to AG05836B-15 (66%) and H9 (47%). Bar graphs showing average and error bars represent ± SD of at least three independent differentiation runs (except MLY7 for H9 which is represents a single differentiation run). (**B**) Cardiomyocytes derived from hESC-H1and hiPSC lines (Detroit 551-A and AG05836B-15) were collected on Day 15 of differentiation and stained with either surface (CD144-FITC and CD140b-PE) or intracellular markers (TNNT2). Cells were stained first to distinguish live/dead cells and then co-stained with conjugated antibodies against surface markers including CD144-FITC and CD140b-PE or stained for the intracellular marker, TNNT2. The H1-derived cardiomyocyte culture with 74.5% TNNT2^+^ cells contained 9.5% CD140b positive and 3.7% CD144 positive cells. In the Detroit 551-A culture that 77% of the cells were positive for TNNT2, 18% and 5% of the cells were positive for CD140b and CD144 respectively. In addition, while 21% of the cell in AG05836B-15 culture expressed smooth muscle marker, only 2% of the cells expressed endothelial marker. This culture contained 67% TNNT2 positive cells. (**C**) Results of immunofluorescent staining showing positive staining for either the smooth muscle marker ACTA2 (scale bar, 100 μm) or the endothelial cell marker CDH5 (Scale bars, 50 μm). Blue is DAPI and green is Alexa-448 stained ACTA2 (smooth muscle marker) and CDH5 (endothelial cell marker). Pictures were taken using Leica TCS SP5 confocal microscope.

The major cell types formed during cardiac lineage differentiation are cardiomyocytes, smooth muscle and endothelial cells (for review^22^). Co-staining of the cells for markers of committed cardiomyocytes (TNNT2 and MYL7) at later stages of differentiation (Day12-19) revealed that 82 ± 7% and 60 ± 10% of the cells from the H1 culture were positive for these markers whereas 85.7 ± 3% of the Detroit551-A cells were positive for TNNT2 and 79.7 ± 3% of them were positive for MYL7 (Fig. 5A). Based on flow cytometric results, H9 had the lowest differentiation efficiency with 47 ± 13% TNNT2-positive and 45% MYL7- positive cells. We then stained cardiomyocytes for CDH5 (a marker for the endothelial lineage) and ACTA2 (smooth muscle) on day 15. This analysis confirmed the presence of smooth muscle and endothelial cells, which are expected to arise as a by-product during cardiac lineage differentiation (Fig. 5C).

We used flow cytometry to investigate the relative proportions of endothelial and smooth muscle cells in cardiac cultures on Day15^23^. The surface markers CD144 and CD140b were used to identify endothelial and smooth muscle respectively. Parallel, samples from the same 96 well plate were stained with an internal cardiomyocyte marker, TNNT2 to provide an estimate of the number of cardiomyocytes (Fig. 5B). In the H1-derived cardiomyocyte culture 74.5 ± 8% were TNNT2 positive cells, 9.5 ± 2.94% stained for the smooth muscle marker (CD140b) and only 3.71 ± 0.81% of the population expressed the endothelial marker (CD144) (Fig. 5B). In the Detroit 551-A culture, the proportion of TNNT2 positive cells was 77 ± 0.18% while 18.45 ± 0.1% were CD140b positive cells and 5.53 ± 0.02% were endothelial cells. In the AG05836B-15 culture 67.2 ± 0.2% were TNNT2 positive cells, while 21 ± 0.1% and 2 ± 0.007% of the cells were positive for CD140b and CD144 markers respectively (Fig. 5B).

#### Investigating the inter-well heterogeneity

**Figure 6:**
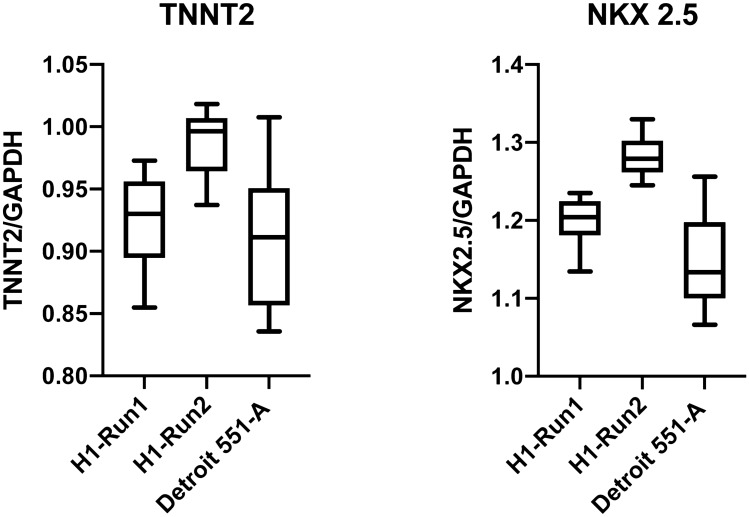
Investigation of inter-well heterogeneity across a 96 well plate and different runs of differentiation. The boxplot analysis shows the variation in expression of two cardiomyocyte markers, *TNNT2* and *NKX2-5* related to the house keeping gene *GAPDH* in the ES line H1 and the iPSC line Detroit 551-A. Cells in both runs were > 25 days of differentiation. Run 1 comprises 12 separate well samples collected from one plate of differentiation of H1 while Run 2 comprises 12 samples taken from 3 different plates of one round of H1 differentiation. The Detroit cell samples are collected from 3 different plates of one run of differentiation.

In order to investigate the heterogeneity between wells, we extracted RNA from multiple wells and performed qPCR to assess the expression of *TNNT2* and *NKX2-5* relative to a housekeeping gene *GAPDH*. We selected up to twelve wells of a differentiation run at random and isolated total RNA using the MagMAx isolation kit. We looked at 2 differentiation runs of H1 and from the first analysed 12 wells of one plate while on the second run we analysed multiple wells of 3 different plates. We also isolated total RNA of multiple wells from 3 different plates of one run of Detroit 551-A differentiation. The data is shown in supplementary table S1online. The variation is shown in Fig. 6 and we calculated the coefficient of variation between these CT values, which varied between 1.96 and 7.30% confirming that there were similar numbers of cardiomyocytes in each well.

### Electrophysiological validation of hiPSC-derived cardiomyocyte

**Figure 7:**
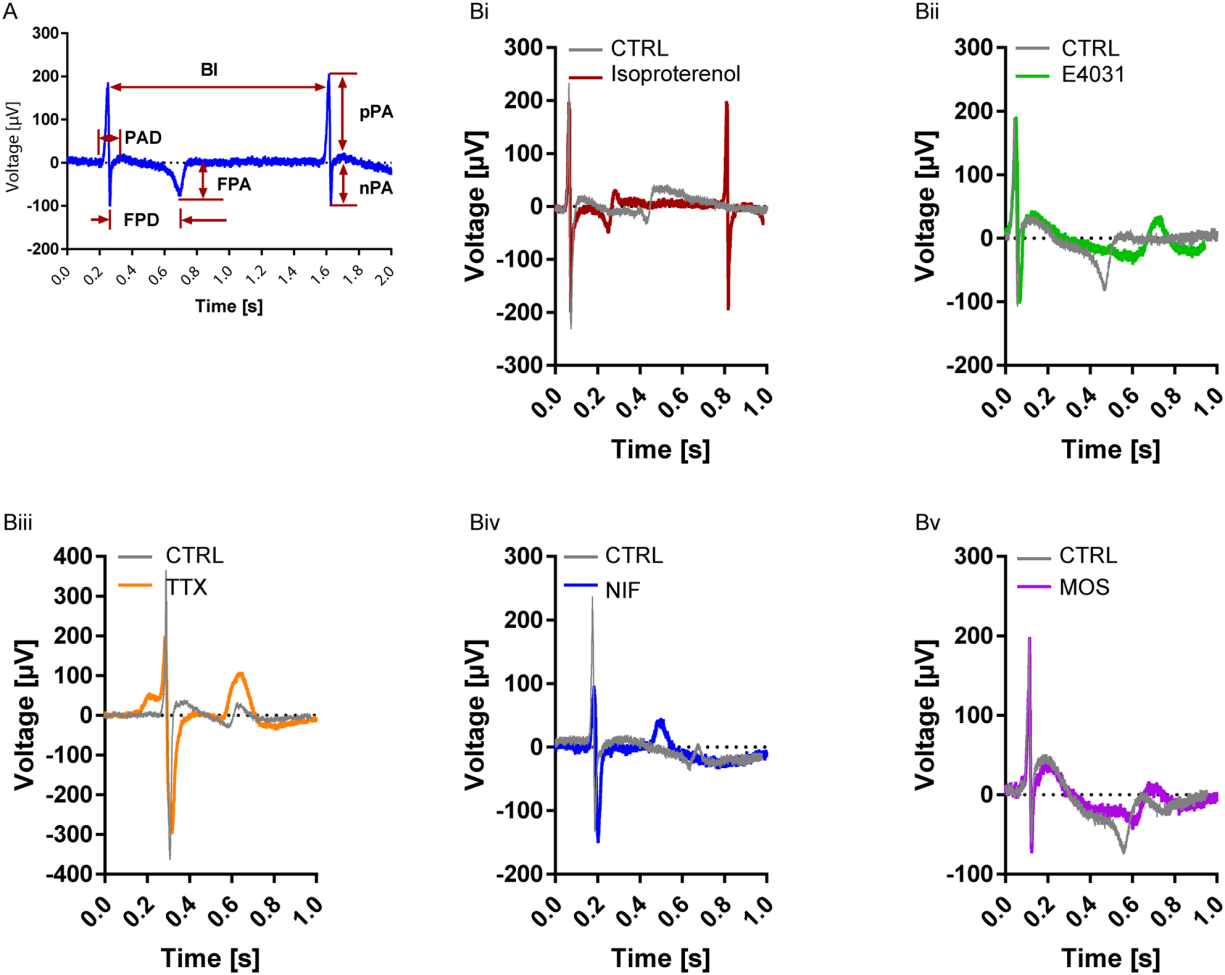
MEA Recording of hiPSC-derived cardiomyocytes. (**A**) Representative trace recorded with the MEA showing the analysis parameter to evaluate the functionality of hiPSC-derived cardiomyocytes. R/Q and T peak; field potential duration (FPD), field potential amplitude (FPA), Q/R peak differentiated into positive peak amplitude (pPA) and negative peak amplitude (nPA), beat interval (BI); (**B**) Representative the electrophysiological properties of H1-derived cardiomyocytes before (no-drug) and after applying (i) β_1_ and β_2_ adrenoreceptor agonist Isoproterenol (ISO), (ii) potassium channel antagonist E403, (iii) sodium channel antagonist tetrodotoxin (TTX), (iv) L-type calcium channel antagonist Nifedipine (NIF) and (v) 5-Hydroxytryptamine (serotonin) receptor -HT4 agonist and HT3 antagonist Mosapride (MOS). The shaded area represents the field potential duration (FPD), also known as QT interval, measured during analysis. Electrophysiological parameters were analyzed and visualized using the analysis tools Analyzer and Spike sorter in the MC_Rack software (Version 4.6.2).

Cardiac function including rhythmicity and contractility depend on the expression of number of ion channels such as sodium, potassium and calcium channels. To test whether the differentiated cardiomyocytes had proper electrophysiological properties, we employed Microelectrode arrays, MEA, and challenge them with different cardiac drugs. Microelectrode arrays provide a highly sensitive, non-invasive method for studying physiological properties of electrically active cells. MEA records electrical waveform signals that are called extracellular field potentials (FPs) and which are generated and shaped by monolayers or small clusters of cardiomyocytes. FP contour represents the cardiac action potential and, reflects to some extent the electrocardiogram recording. Typically, one sees a rapid upstroke that corresponds to the Na^+^ influx (R/Q peak) and membrane depolarization, a slow wave/plateau phase thought to correspond to the Ca2^+^ influx, and a repolarization phase corresponding to a predominant K^+^ efflux (T peak) (Fig. 7A).

We transferred H1 and Detroit551-A line-derived cardiomyocytes into the MEA chamber on day 11–13 of differentiation and monitored the steadiness and beat consistency signatures up to 14 days (Day 12–26 of differentiation). The period of observation was selected based on the previous studies suggesting the best stage of differentiation for transferring the cardiomyocytes to MEA chamber for checking their electrophysiological activities^5,24^.

H1-derived cardiomyocytes showed an average FP duration of 298 ± 20 ms, and a beat interval (BI) of 42 ± 2 beats/min, whereas Detroit551-A-derived cardiomyocytes showed an average FP duration of 75 ± 5 ms, and a BI of 49 ± 4 beats/min, respectively. Beating interval became more irregular after 26 days in culture as has been previously described^5^. To test functionality of the hPSC-derived cardiomyocytes, the following drugs were applied—β_1_ and β_2_ adrenoreceptor agonist isoproterenol (1 µM), potassium channel antagonist E4031 (1 µM), sodium channel antagonist tetrodotoxin (TTX, 5 µM)), L-type calcium channel antagonist nifedipine (5 nM), and the 5-hydroxytryptamine (serotonin) receptor -HT4 agonist and HT3 antagonist Mosapride (350 nM) (Fig. 7Bi-v, Table 1). While a more limited number of drugs were applied to Detroit 551-A, these cells exhibited the same alterations in their electrophysiological pattern following the application of isoproterenol and E4031.

**Table 1:** Electrophysiological properties of H1- and Detroit551A-derived cardiomyocytes.

Drugs	CM line	Exp	Groups	BI (beats/min)		FPA (µV)		FPD (ms)		FPUS (µV/ms)		pPA (µV)	
ISO 1 µM	H1	n_C_ = 6; n_R_ = 11;	CTRL_ISO_	47 ± 3	*P* < 0.0001****	66.5 ± 11.6	*P* = 0.2792	315 ± 22	*P* < 0.0001****	0.25 ± 0.05	*P* = 0.0139*	245.3 ± 27.7	*P* = 0.3884
			ISO	78 ± 3		70.7 ± 10.2		183 ± 14		0.44 ± 0.07		264.1 ± 30.2	
	Detroit 551-A	n_C_ = 3; n_R_ = 4;	CTRL_ISO_	44 ± 8	*P* < 0.0001****	48.5 ± 5.8	*P* = 0.3548	66 ± 8	*P* = 0.1099	0.95 ± 0.13	*P* = 0.1514	125.9 ± 29.0	*P* = 0.1439
			ISO	64 ± 7		54.3 ± 5.9		57 ± 8		1.41 ± 0.32		81.0 ± 10.6	
E4031 1 µM	H1	n_C_ = 6; n_R_ = 10;	CTRL_E4031_	50 ± 2	*P* = 0.0046**	70.9 ± 7.6	*P* < 0.0001****	302 ± 18	*P* < 0.0001****	0.25 ± 0.03	*P* < 0.0001****	165.9 ± 19.8	*P* < 0.0001****
			E4031	42 ± 1		36.9 ± 4.0		651 ± 47		0.07 ± 0.01		113.4 ± 18.5	
	Detroit 551-A	n_C_ = 3; n_R_ = 3;	CTRL_E4031_	77 ± 1	*P* = 0.0078**	44.3 ± 6.4	*P* = 0.1294	57 ± 6	*P* = 0.0078**	0.74 ± 0.11	*P* = 0.0078**	292.9 ± 33.6	*P* = 0.0078**
			E4031	66 ± 1		32.4 ± 3.7		143 ± 21		0.28 ± 0.02		143.6 ± 13.8	
TTX 5 µM	H1	n_C_ = 6; n_R_ = 12;	CTRL_TTX_	41 ± 2	*P* < 0.0001****	79.5 ± 7.6	*P* = 0.0033**	353 ± 18	*P* = 0.0622	0.25 ± 0.03	*P* = 0.4393	303.1 ± 27.0	*P* < 0.0001****
			TTX	25 ± 2		109.4 ± 11.0		394 ± 23		0.35 ± 0.05		147.4 ± 20.4	
	Detroit 551-A	n_C_ = 3; n_R_ = 3;	CTRL_TTX_	46 ± 4	*P* = 0.0137*	48.5 ± 12.1	*P* = 0.1250	45 ± 4	*P* = 0.0625	0.76 ± 0.11	*P* = 0.0122*	253.1 ± 25.2	*P* = 0.0005***
			TTX	33 ± 7		67.3 ± 13.2		40 ± 3		1.35 ± 0.32		123.6 ± 13.6	
NIF 5 nM	H1	n_C_ = 6; n_R_ = 10;	CTRL_NIF_	45 ± 2	*P* = 0.0044**	59.3 ± 8.7	*P* = 0.1796	361 ± 25	*P* < 0.0001****	0.19 ± 0.04	*P* = 0.0066**	160.1 ± 25.4	*P* = 0.0027**
			NIF	36 ± 2		69.9 ± 12.0		195 ± 10		0.36 ± 0.06		111.3 ± 15.9	
MOS 350 nM	H1	n_C_ = 6; n_R_ = 11;	CTRL_MOS_	46 ± 2	*P* = 0.0067**	39.8 ± 3.1	*P* = 0.0110*	325 ± 26	*P* = 0.0562	0.14 ± 0.02	*P* = 0.0039**	137.7 ± 20.9	*P* = 0.0562
			MOS	52 ± 2		25.4 ± 2.1		387 ± 30		0.08 ± 0.01		127.6 ± 18.5	

*ISO* Isoproterenol, *TTX* Tetrodotoxin, *NIF* nifedipine, *MOS* mosaprid, *BI* beat interval, *FPA* field potential amplitude, *FPD* field potential amplitude, *FPUS* field potential amplitude upstroke speed, *pPA* positive peak amplitude, *n*_*C*_ numbers of independent cultures, *n*_*R*_ numbers of independent recordings, statistic: mean ± SEM, paired-nonparametric Wilcoxen signed rank test.

As expected, isoproterenol increased the beating frequency and led to a significant reduction of the FP duration (FPD, Fig. 7Bi, Table 1). E4031 reduced the FP amplitude (FPA, Fig. 7Bii, Table 1) as expected, but contrary to other reports, we observed an increased in the FP duration (FPD) and a minor prolongation of the beat interval^5,24^. Application of TTX led to significant reduction of the positive peak amplitude (pPA), increased FPA, prolonged BI, but no effect on FPD (Fig. 7Biii, Table 1). Nifedipine, behaved as described by reducing the pPA and FPD with no effect on the BI (Fig. 7Biv, Table 1). Mosapride^25,26^ impacted the FPA and led to a notable reduction of the FPA/FPD ratio, also called field potential amplitude upstroke speed (FPUS), an indicator of sodium and L-type calcium channel function^27^ (Fig. 7Bv, Table 1).

## Discussion

The aim of this study was to develop a scaled down platform for cardiac differentiation that was amenable for high throughput screening, efficient and reproducible without any genetic modification or additional enrichment processes, while at the same time reducing overall cost. We modified the monolayer-based, small molecule approach described by Lian and colleagues^8^ to achieve these goals. We replaced 12-well plate with 96-well plate format, mTeSR1 medium with the simpler Essential 8 formulation and substituted Matrigel with the cheaper and more defined extra cellular matrix, Geltrex at the undifferentiated hPSC expansion stage. This combination of modifications reduced the cost of differentiation while still yielding high levels of TNNT2 positive cells at later stages of differentiation.

The impact of confluency on differentiation has been addressed^12,18^ and we also showed that the yield of differentiated cardiomyocytes was highly dependent on hPSC colony density (Fig. 2, Supplementary Fig. S1). Thus, the 12-well format produced the best yield at a confluency of 85–90%^7,28^, whereas in 96-well plates, we found that a confluency of 60–70% gave excellent numbers of cardiomyocytes (H1 82%, Detroit551-A 85%). Further, we observed that CHIR99021 had to be adjusted to altered confluency^10,29^ and that the concentration was dependent on the cell type used for differentiation. Cardiomyocytes derived from the hESC lines H1 and H9 required a higher CHIR990201 concentration than those derived from hiPSCs (Detroit 551-A and AG05836B-15) to achieve an optimal differentiation yield. The yield of cardiomyocytes did vary between the two hiPSC lines (85% for Detroit 551-A and 66% for AG05836B-15). This might be due to the different reprogramming systems used (Detroit 551-A—retroviral reprogrammed, AG05836B-15 integration free Sendai virus reprogramed), but we did not investigate this in detail.

Moreover, we assessed the expression of key genetic markers in the cultures to validate the developmental trajectory towards a cardiomyocyte fate (Fig. 3, Supplementary Fig. S2 and Fig. S3A). ISL-1 is one of the key markers for cardiac progenitors and it has been shown that ISL-1 expression levels gradually decline in committed cardiomyocytes after its rise at the progenitor stage (day 5)^30,31^. Of note, H1 followed this pattern and resulted in a high level of TNNT2 + (more than 80%), while the expression level of ISL-1 remained high throughout the H9 differentiation resulting in 47% TNNT2 positive cells (Supplementary Fig. S3A). Our findings, which are similar to previous reports showing the poor differentiation capacity of H9 toward cardiomyocytes, may be explained by the inability to reduce expression of ISL-1 after the progenitor stage^23^. Correct expression patterns of mature cardiomyocytes markers such as *HOPX, MYH6, MYH7* and *NKX2.5* on D30 of differentiation confirmed the maturity of the cardiomyocytes generated in this 96-well microplate format (supplementary Fig. S4)^6^.

Cardiovascular progenitors are able to develop into cardiomyocytes, endothelial cells, cardiac fibroblasts and smooth muscle cells. Using our protocol, we demonstrated that between day 10 and 15 the majority of hPSC derived cardiomyocytes stained for TNNT2 as well as MYL7 (Fig. 5A). We also investigated the non-cardiomyocyte populations generated using our protocol. The number of TNNT2 positive varied between 64 ± 8% for hESC and 76 ± 8% for hiPSC. Differentiation of H1 resulted in 3–4% endothelial cells and 9–11% smooth muscle cells. The percentage of endothelial cells in hiPSC-derived cultures was higher than in ES derived cultures with 18.45% in Detroit 551-A and 21% in AG05836B-15, smooth muscle cell numbers were lower in the Detroit 551-A and AG05836B-15 hiPSC lines compared to H1 (Fig. 5B).

In order to ensure that each well of the 96 well plate provided a similar complement of cells, an important factor if we are to use this method for e.g. drug screening, we investigated the well-to-well heterogeneity in our cultures. We used both immunocytochemistry and RT-qPCR to determine the relative amount of cardiomyocytes in each well. We used microscopy to assess the homogeneity of the cardiomyocyte population and found that the cultures contained high numbers of TNNT2 positive cells at day 10 (Fig. 4). We also studied the composition using PCR to identify the cardiac specific marker TNNT2 and NKX2-5 (Fig. 6). This assay suggested that there was a low heterogeneity between the wells of one run of differentiation and between two separate runs of differentiation. These results demonstrate that this smaller format method produces reproducible cardiomyocyte populations both within a single plate and in different runs of differentiation.

Our electrophysiological data (Fig. 7, Table 1) are in line with previous publications and confirm that the cardiomyocytes produced by this method are physiologically functional. Beating rate increased in both, cardiomyocytes derived from H1 and Detroit 551-A following addition of the β-adrenergic agonist isoprenaline, known to have a positive chronotropic effect on hPSC-derived cardiomyocytes^32,33^. Our cardiomyocytes also showed TTX-sensitive and TTX-resistance Na^+^ channels: TTX induced a significant reduction of pPA, without complete elimination as demonstrated previously^34,35^. Furthermore, manipulation of K^+^ efflux by E4031, L-type VGCC dependent calcium influx by Nifidipine or 5-Hydroxytryptamine (serotonin) receptor—HT4/HT3 function by Mesopride showed a clear alterations of cardiomyocyte extracellular FP trace that was consistent with the previous reports^5^. These findings suggest that our established 96-well format in vitro system recapitulates the functional characteristics of cardiomyocytes.

Several approaches to enrich the cardiomyocyte population have been described including genetic-modification (by the expression of MYH6), using surface protein as a specific marker for cardiomyocytes (signal-regulatory protein alpha) or selection based on targeting highly active mitochondria using a mitochondrial membrane potential marker (TMRM)^36–38^.

One of the most effective and straightforward approaches appears to be the metabolic selection using lactate instead of glucose to force the cells to use their respiratory chain instead of glycolysis and selecting cardiac cells based on their energy profile^28,39,40^.

Since we are interested in studying disease mechanisms, one key aim of our study was to produce a high number of cardiomyocytes without over manipulation, as this may have unwanted cellular and molecular effects. We did not therefore investigate the use of these additional measures. Our protocol results in a high percentage of cardiomyocytes that express the cardiac marker *TNTN2* in both hESCs and hiPSCs without the use of any purification method. In principle, however, any of these methods could be combined with our protocol to further enhance purification.

Although, well-defined differentiation conditions have greatly improved reproducibility of differentiation, inter-experimental reproducibility still remains a key challenge^12^. The strength of this protocol is the 96-well format, which allows inter-experimental comparability, thus potentially reducing inter-experimental variability and increasing the precision of reported results. Further, it offers the possibility of combining differentiated cells from different wells or pooling data from individual wells of the 96-well format to balance out the inter-experimental variation and improve reproducibility. This protocol also demonstrates it is not necessary to first differentiate the cells in larger format before reseeding them in to 96 wells for experimentation such as high throughput analysis^41,42^.

## Conclusion

Here we have demonstrated the efficient differentiation of cardiomyocytes in a 96-well microplate format, that reduces both workload and cost, improving both consistency and precision. This platform provides an adaptable system for monitoring the differentiation and development of cardiac tissue from very early stages all the way to functional tissue in 96-well format and is well suited to the investigation of early disease development.

## Material and methods

### hiPSC generation and characterization

H1 and H9 were obtained from WiCell Research Institute (Madison, Wisconsin, WiCell) and well characterized as previously described^1^. Detroit 551-A fibroblasts were obtained from ATCC (American Type Culture Collection), AG05836B-15 fibroblasts were obtained from the Coriell Cell Repositories and reprogramed into hiPSCs and characterized as previously described in Mathipathi et al.^19,20^.

### Cardiomyocyte differentiation

Geltrex (#A1413302, Thermo Fisher Scientific) was diluted 1:100 in ice cold Advanced DMEM/F-12 (#12634010, Thermo Fisher Scientific) to coat wells in the 96-well plate (85 µl/well). Coated plates were incubated at 37 °C for at least 30 min before use. Before plating the hiPSC cells, colonies were broken down to single cells after treating with 0.5 mM EDTA to have a homogeneous cell suspension. Human iPSCs were seeded in Geltrex coated wells at a density of 2.4 × 10^4^ cells/cm^2^ using Essential 8 Medium (E8) (#A1517001, Thermo Fisher Scientific) supplemented with 10 µM Rock inhibitor (Y27632; #1254, Tocris Bioscience) for 24hrs. After 24hrs, the medium was changed with freshly prepared E8 medium without Y27632 and medium was exchanged every day. After two or three days, when the confluency of the culture reached 60–70%, cells were treated with 6 µM (optimal concentration for hiPSC) or 8 µM (optimal concentration for hESC) of CHIR99021 (#4423, Tocris Bioscience) in RPMI 1640 (#61,870, Thermo Fisher Scientific) supplemented with B27-without insulin (#A1895601, Thermo Fisher Scientific). After 24 h the medium containing CHIR99021 was changed to RPMI-B27 without insulin alone and left for 48 h. On day 3 cells were treated with 5 µM IWP2 (#3533, Tocris Bioscience) diluted in RPMI-B27 without insulin and incubated for 48 h. On day 5 medium was changed to freshly prepared RPMI-B27 without insulin for 48 h. At day 7 medium was changed to RPMI-B27 with insulin (#17,504,044, Thermo Fisher Scientific) without any extra supplement and medium was changed every two days thereafter. Schematic representation of the protocol (Fig. 1A). Working volume in all the steps of the protocol was 200 µl per well of 96-well plate.

### Gene expression via real-time PCR

RNA used for gene expression studies was isolated by MagMAX-96 Total RNA Isolation Kit (#AM1830, Thermo Fisher Scientific). Cells were lysed using lysis buffer supplemented by the kit directly inside the plate after a quick wash with PBS. Lysate was either processed immediately for RNA isolation using an automated MagMAX express 96 or stored in—80 °C for later extraction. cDNA synthesis and Real-time qPCR were performed using EXPRESS One-Step Superscript qRT-PCR Kit (#11781-01 K,Thermo Fisher Scientific) and TaqMan probes (see Supplementary Table S2 online). Applied Biosystems 7500-Fast real-time PCR System (Thermo Fisher Scientific) was used to perform qPCR. All qPCR reactions were performed in triplicate and normalized to the geometric mean of *ACTB* and *GAPDH* as endogenous control genes, and assessed using the comparative ΔΔCt method by normalizing differentiated cells to undifferentiated pluripotent stem cells. Results are shown as the mean of three independent differentiation runs and error bars represent standard deviation of the mean.

### Fluorescent microscopy

Cells of single well of 96 well plate were seeded either on Geltrex coated glass coverslips or in Geltrex coated Millicell EZ SLIDES (#PEZGS0816, Merck Millipore). Cells were fixed with 4% paraformaldehyde (#43368, Alfa Aesar) for 10 min at room temperature and permeabilized with 0.3% Tween 20 (#822184, Merck Millipore) diluted in PBS for 1 hr at room temperature. All the primary and secondary antibodies were diluted in blocking buffer consisting of 0.3 M glycine, 5% goat serum and 1% BSA in PBS. Final concentration of 10 µg/ml was used for all the primary antibodies and incubated overnight at 4 °C. Alexa Fluor 488 and 568 (Thermo Fisher Scientific) was used as secondary antibodies and were diluted in blocking buffer (1:1000) and incubated for 1 h at room temperature. Nuclei were stained with Gold Antifade Reagent with DAPI (#P36935, Thermo Fisher Scientific). Confocal microscopy images were taken by either Zeiss LSM 510 META or Leica TCS SP5 at Molecular Imaging Center (MIC), University of Bergen, and data analysis and image editing were done with Fiji^43^. Antibodies are listed in Supplementary Table S2 online.

### Flow cytometry

#### Sample preparation for detecting extracellular antigens; surface markers

Cells were dissociated into single cells using 100 µl TrypLE Express Enzyme (Thermo Fisher Scientific) for 20 min at 37 °C and after a quick wash with PBS. Cells were collected after adding 100 µl of culture medium (e.g. RPMI B27 +) supplemented with 20% FBS and each eight wells of 96-well format were pooled and filtered using 40 μm pre-wet cell strainer (# 431750, Sigma). Each sample counted prior to spinning down at 400 g for 5 min at RT.

Cell pellet was resuspended in Running buffer consisting of autoMACS Rinsing Solution (#130-091-222, Miltenyi Biotec) and MACS BSA Stock Solution (# 130-091-376, Miltenyi Biotec)-final concentration of BSA was 0.5%. Number of cells in each sample was adjusted for 10^5^ cells/100 μl. Cells were stained with Dead cell Discriminator (1:50), provided by the Fixation and Dead Cell Discrimination Kit (#130-091-163, Miltenyi Biotec) and incubated on ice while exposing to a 60 W light bulb for 10 min. Subsequently, the optimal concentration of conjugated antibodies for flow cytometry reported by the supplier for each batch was used. Cells were incubated for 10 min at 4 °C in the dark with pre-conjugated antibodies; CD144- FITC and CD140b-PE. Cells were washed by adding 1 ml of running buffer per 10^5^ cells and spinning down at 300 g for 10 min at RT. Cell pellet was resuspended in 300 μl of running buffer and cells were fixed by adding 150 μl of FiX solution and 5 μl of Discriminator Stop Reagent provided by the Fixation and Dead Cell Discrimination Kit. Fluorescence minus one control was used to adjust the gate for positive cells, due to the co-staining for live/dead staining and different markers within one sample. Proper Isotype controls and compensation beads were also included for gating (Supplementary Fig. S5). At least 30,000 events were collected for each sample with a 100 µm nozzle by a Sony cell sorter SH800 (Sony Biotechnology Inc.) and data were analyzed and visualized using FlowJo V.10.5.0 (FlowJo LLC, OR, USA) software (www.FlowJo.com). Antibodies are listed in Supplementary Table S2 online.

#### Sample preparation for detecting the intracellular antigens

Cells were washed in DPBS (#14190250, ThermoFisher) three times and dissociated into single cells using Enzyme T diluted in Buffer X (1:10) provided by Multi Tissue Dissociation Kit 3 (#130-110-204, Miltenyi Biotec) for 10–15 min at 37 °C. Equal volume of culture medium (e.g. RPMI B27 +) supplemented with 20% FBS was added to each well and each eight wells of 96-well format were pooled and filtered using 40 μm pre-wet cell strainer (# 431750, Sigma). Cells were counted prior to spinning down at 400 g for 5 min at RT. Cell pellet was resuspended in InsideFix solution, provided by Inside Stain Kit (#130-090-477, Miltenyi Biotec), diluted in DPBS (1:1) and incubated for 10 min at RT. Number of cells in each sample was adjusted for 10^5^ cells/100 μl. Cells were washed by adding 1 ml of running buffer per 10^5^ cells and spinning down at 300 g for 10 min at RT. Cell pellet was resuspended in Inside Perm solution—a component of the Inside Stain Kit- and stained with recommended dilution of pre-conjugated antibodies for 10 min in the dark at RT. Antibodies were pre-conjugated with Fluorescein isothiocyanate (FITC). Cells were washed with 1 ml Inside Perm solution per 10^5^ cells for 5 min at 400 g. Cell pellet was resuspended in 300 μl of running buffer and proceed to analysis. Fluorescence minus one control was used to adjust the gate for positive cells (Supplementary Fig. S5). At least 30,000 events were collected for each sample with a 100 µm nozzle by a Sony cell sorter SH800 (Sony Biotechnology Inc.) and data were analyzed and presented using V.10.5.0 (FlowJo LLC, OR, USA, www.FlowJo.com) software. Antibodies are listed in Supplementary Table S2 online.

NOTE: Adherent cardiomyocytes generated in 96 well plate were dissociated into single cells by using TrypLE Express enzyme for detecting extracellular antigens and using Multi Tissue Dissociation Kit 3 for detecting intracellular antigen by flow cytometry. The difference between the efficiency of these two methods was not significant (*P *value = 0.17) for dissociating cardiomyocytes. Dissociation of cardiomyocytes in to single cell with Multi Tissue Dissociation Kit 3 was faster specifically for dissociating more mature culture, however it caused changes in extracellular antigens structure that could not be detected by antibodies and subsequently, no detectable signal for flow cytometric analysis. Therefore, we used TrypLE Express enzyme for detection of extracellular antigens.

### Microelectrode array measurements

Human Pluripotent stem cells including H1 and Detroit 551-A were differentiated towards the cardiomyocytes as describe in cardiomyocyte differentiation section. At day 11–14 of differentiation, 1 × 10^6^ cells of cardiomyocyte culture from eight wells of a 96-well plate were pooled, and transferred into a single chamber microelectrode array (MEA) unit containing 60 integrated TiN electrodes (#60MEA200/30iR-Ti-gr; Multichannel Systems).

To dissociate the cardiomyocytes into single cells or small cell clump of 10–12 cells, cells were quickly washed with DPBS and incubated with TRYPLE Express Enzyme (Thermo Fisher Scientific) at 37 °C for 10–15 min (100 μl per well). TRYPLE Express Enzyme was neutralized with the equal volume of RPMI-B27 with insulin (#17504044, Thermo Fisher Scientific) supplemented with 20% FBS. Collected cells from each well were transferred into a falcon tube and washed with RPMI-B27 containing insulin and 20% FBS. Cells suspension was centrifuged at 400 g for 5 min at RT and cell pellet was gently resuspended in 1 ml of RPMI-B27 with insulin supplemented with 10 μM of Y27632 (#1254, Tocris Bioscience) and plated in MEA chamber that was pre-coated with Geltrex, 1:100 diluted in Advanced DMEM/F-12, for at least 30 min at 37 °C. After 12 h of incubation at 37 °C with 5% CO_2_, Cardiomyocytes were anchored over the electrode field using a 0.8 g glass coated steel ring covered with a nylon mesh (#ALA HSG-MEA-5BD; Multichannel systems). After observing spontaneous beating of the cell accumulate 48 h post transferring, field potential (FP) recordings were commenced. At a sampling rate of 20 kHz local field potentials at each electrode were collected over a period of 15 min at 35 °C using the MEA2100-HS2 × 60 system and the MC-Rack software (Version 4.6.2; Multichannel system, Reutlingen, Germany).

FPs were recorded for 5 min under control conditions, after applying chemical compounds such as selective β-adrenergic agonist Isoprenternol (ISO, 1 µM; #1747; Tocris), K_V_11.1 (hERG) channel antagonist E4031(1 µM; #1808; Tocris), L-type Ca_V_ channel antagonist Nifedipine (NIF, 5 nM; #N-120; Alomone), Na channel antagonist Tetrodotoxin (TTX, 5 µM; #1069; Tocris) and 5-HT3/4 receptor antagonist Mosapride (MOS, 350 nM; #M-225; Alomone) and post-washout.

### Electrophysiological parameter analysis

Using the analysis tools Analyzer and Spike sorter in the MC_Rack software (Version 4.6.2) positive (pPA) and negative peak amplitude (nPA), field potential duration (FPD) and amplitude (FPA) as well as beat interval (BI) were evaluated (Fig. 7A). Field potential amplitude upstroke speed was calculated by dividing FPA by FPD. All data sets are represented as mean ± SD compiling 5 randomly chosen electrodes of at least 5 independent experiments. The chemical compound data were normalized to control for each experiment.

## Supplementary information

Supplementary file1

Supplementary file2

Supplementary file3

Supplementary file4

Supplementary file5

Supplementary file6

Supplementary file7

Supplementary file8
