# Supplementary figures and images for: A method for differentiating human induced pluripotent stem cells toward functional cardiomyocytes in 96-well microplates
Source: Sci Rep. 2020 Oct 28;10:18498. doi: 10.1038/s41598-020-73656-2 (PMC7595118; doi:10.1038/s41598-020-73656-2)

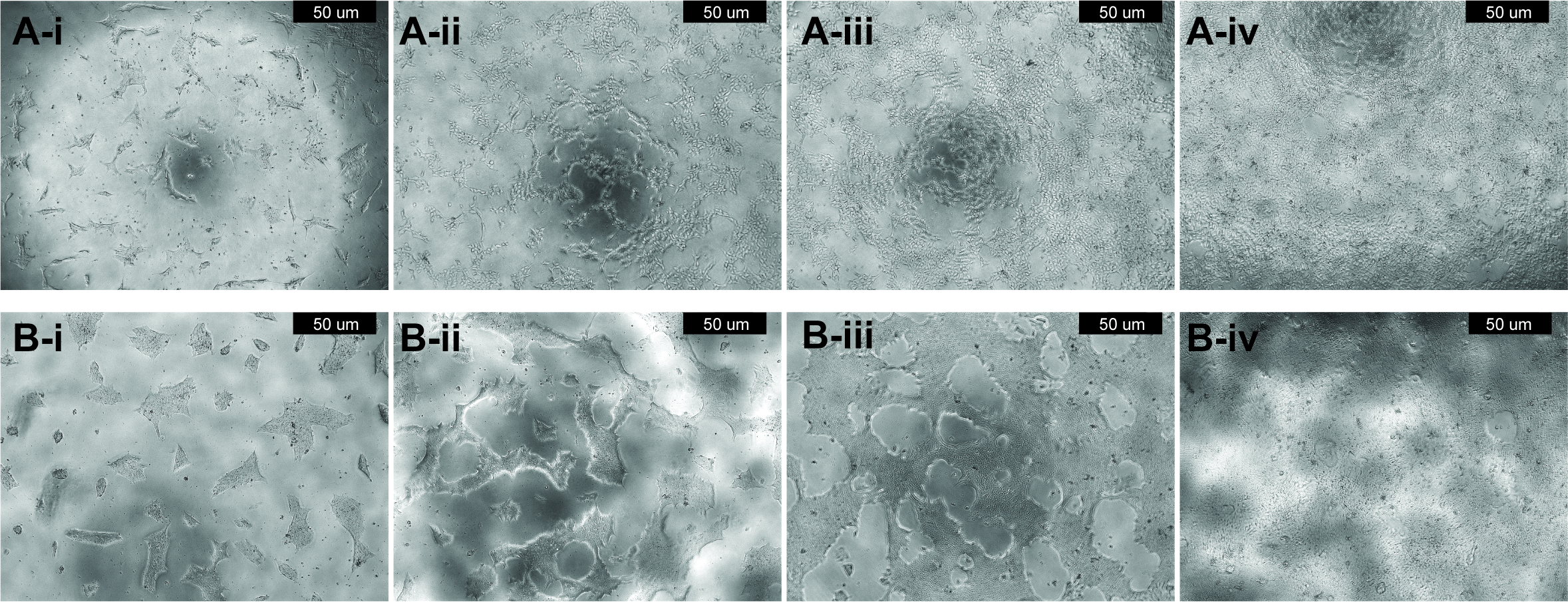

Supplement: Supplementary file 1 — Supplementary file1 [file 41598_2020_73656_MOESM1_ESM.tif]

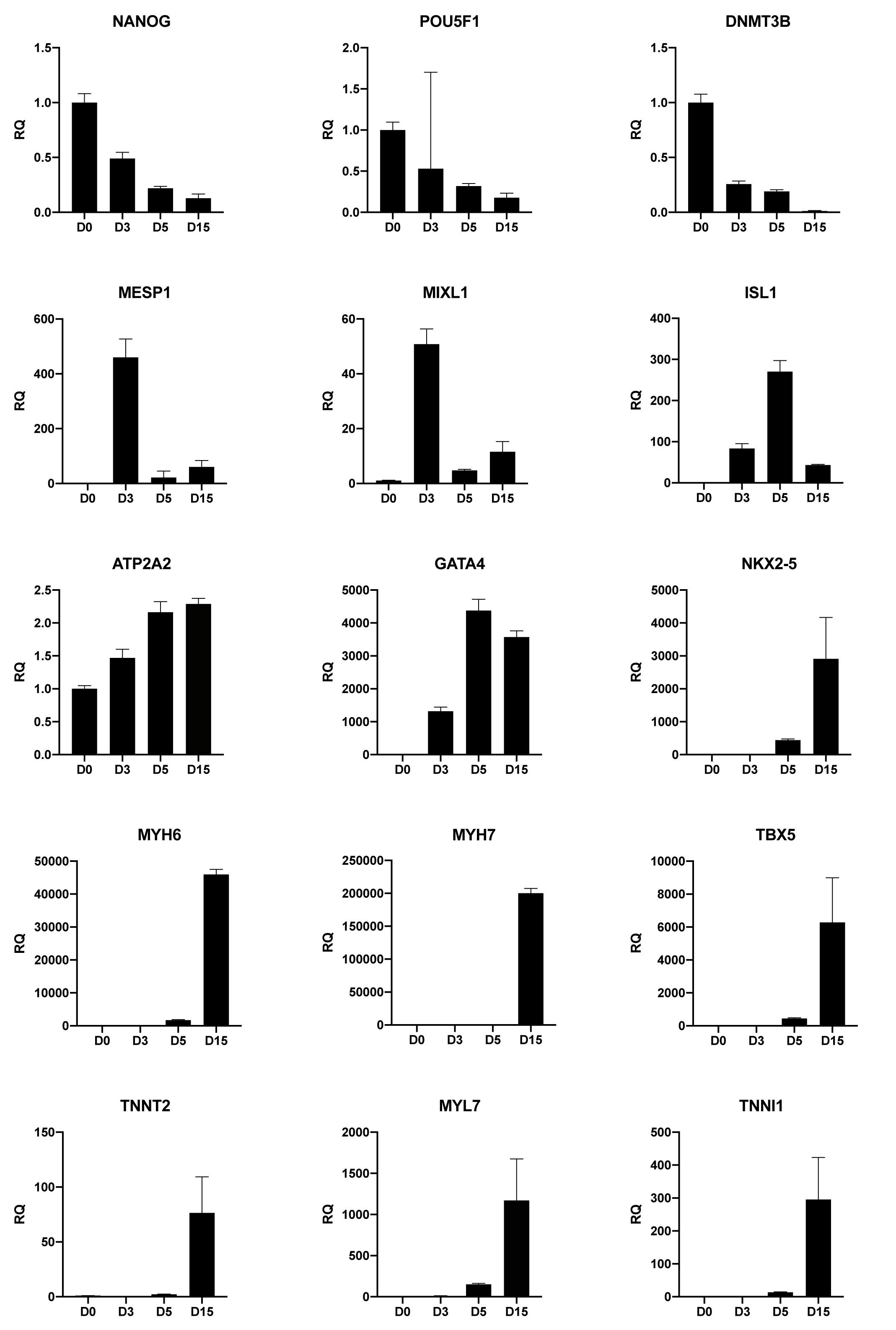

Supplement: Supplementary file 2 — Supplementary file2 [file 41598_2020_73656_MOESM2_ESM.tif]

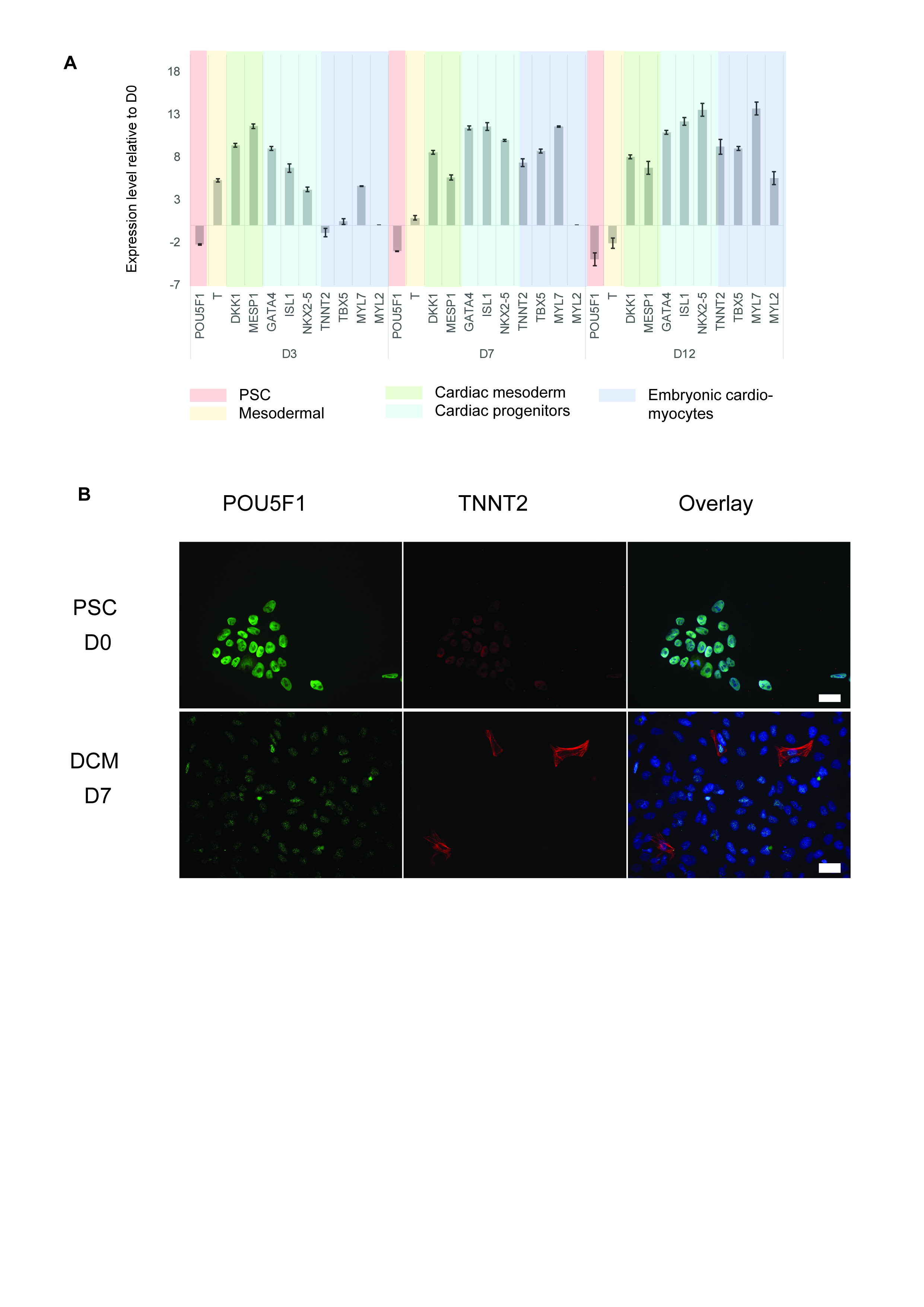

Supplement: Supplementary file 3 — Supplementary file3 [file 41598_2020_73656_MOESM3_ESM.tif]

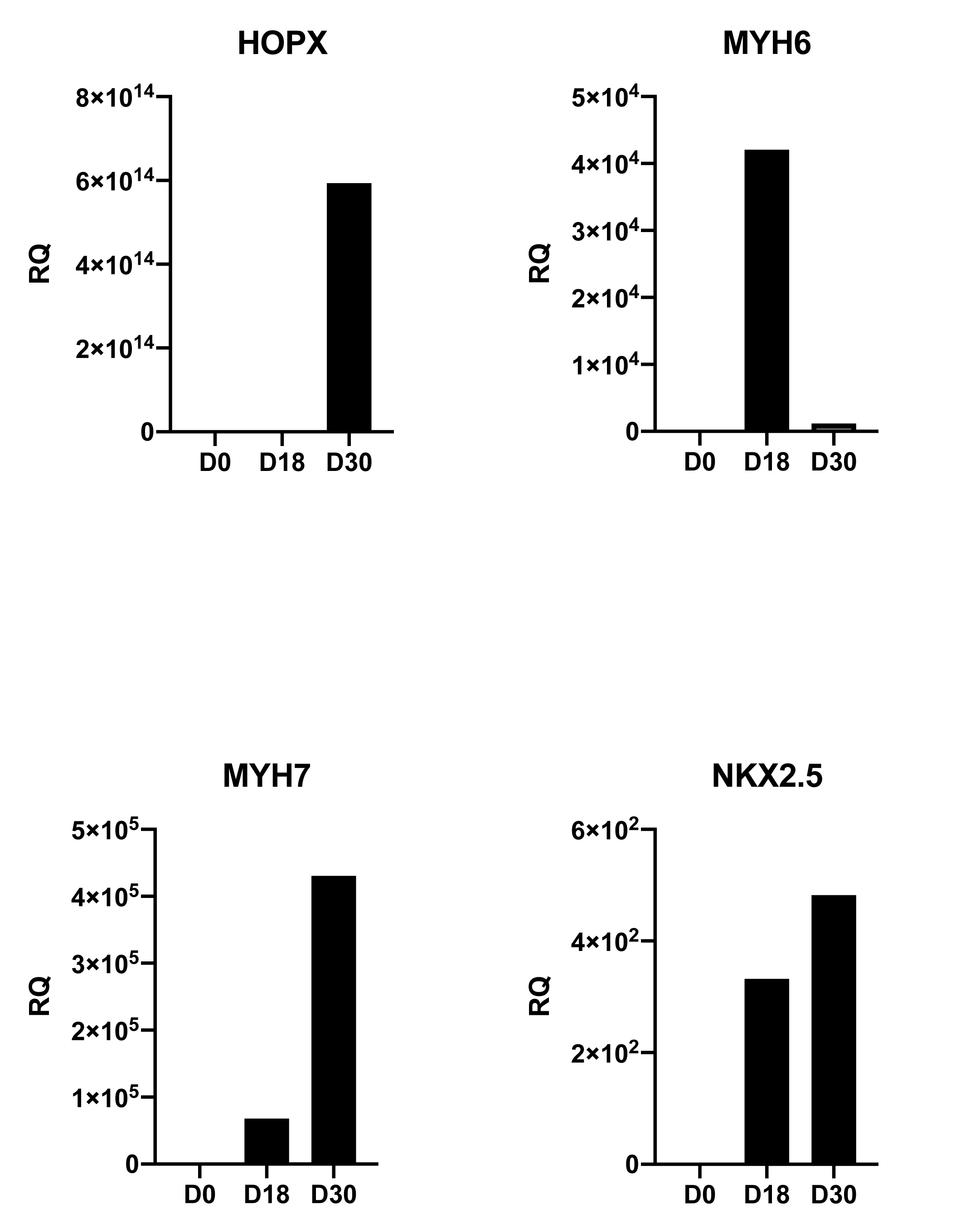

Supplement: Supplementary file 4 — Supplementary file4 [file 41598_2020_73656_MOESM4_ESM.tif]

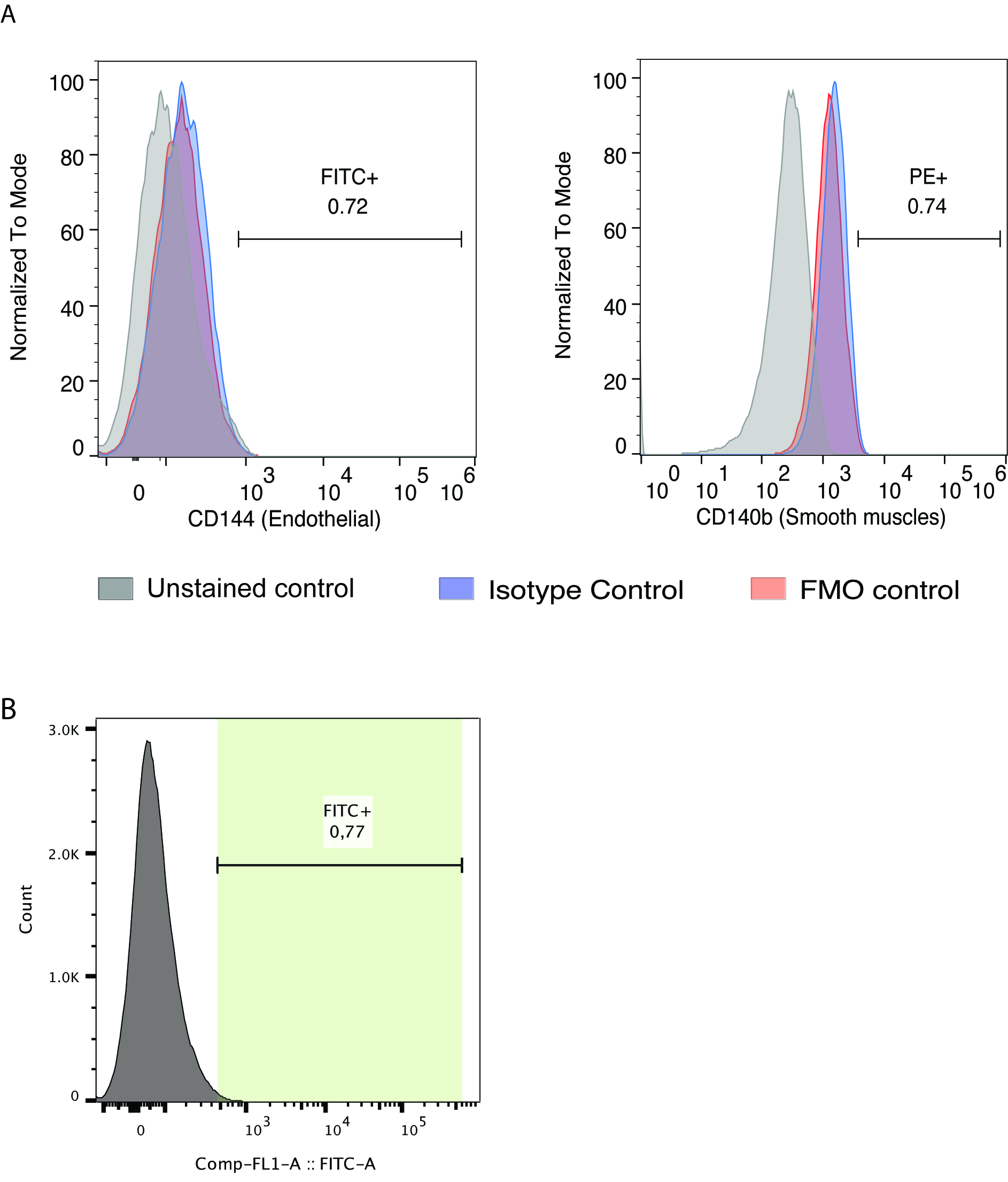

Supplement: Supplementary file 5 — Supplementary file5 [file 41598_2020_73656_MOESM5_ESM.tif]
