# Supplementary material for: A method for differentiating human induced pluripotent stem cells toward functional cardiomyocytes in 96-well microplates
Source: Sci Rep. 2020 Oct 28;10:18498. doi: 10.1038/s41598-020-73656-2 (PMC7595118; doi:10.1038/s41598-020-73656-2)
Supplement: Supplementary file 6 — Supplementary file6 [file 41598_2020_73656_MOESM6_ESM.docx]

SUPPLEMENTARY TABLES

**A method for differentiating human induced pluripotent stem cells toward functional cardiomyocytes in 96-well microplates**

Novin Balafkan, Sepideh Mostafavi, Manja Schubert, Richard Siller, Xiao Liang, Gareth Sullivan, Laurence A. Bindoff

**Supplementary Table S1. Expression of two cardiomyopcyte markers, *TNNT2* and *NKX2-5* in single well of 96 well plate.** The ratio of CT value of *TNNT2* and *NKX2-5* to CT value of the housekeeping gene (*GAPDH*) of the same well was calculated and used for boxplot analysis.

|  | **H1-Run1** | |  | **H1-Run2** | |  | **Detroit- 551-A** | |
| --- | --- | --- | --- | --- | --- | --- | --- | --- |
| **Plate 1** | **TNNT2/**  **GAPDH** | **NKX2-5/**  **GAPDH** |  | **TNNT2/**  **GAPDH** | **NKX2-5/**  **GAPDH** |  | **TNNT2/**  **GAPDH** | **NKX2-5/**  **GAPDH** |
|  | 0.956139536 | 1.015778015 | **Plate 1** | 1.194772754 | 1.302516732 | **Plate 1** | 1.00756806 | 1.25257046 |
|  | 0.972867659 | 1.005521616 |  | 1.206018698 | 1.308690262 |  | 0.98687557 | 1.25630243 |
|  | 0.866792522 | 0.937277184 |  | 1.13410478 | 1.258217263 |  | 0.8955656 | 1.10212642 |
|  | 0.910855933 | 0.96281839 |  | 1.183462783 | 1.283911434 |  | 0.92707995 | 1.14802946 |
|  | 0.854842318 | 0.996865388 | **Plate 2** | 1.055617383 | 1.284905175 | **Plate 2** | 0.92830478 | 1.16168319 |
|  | 1.120783689 | 1.018326948 |  | 1.438343182 | 1.329944629 |  | 0.86026702 | 1.11449094 |
|  | 0.894715841 | 0.968538453 |  | 1.172014938 | 1.274410378 |  | 0.93855131 | 1.17942924 |
|  | 0.971535661 | 1.004025319 |  | 1.222110169 | 1.270765593 |  |  |  |
|  | 0.93476675 | 1.007464663 | **Plate 3** | 1.234872769 | 1.301884353 | **Plate 3** | 0.87943181 | 1.09338735 |
|  | 0.916651164 | 0.939988766 |  | 1.201630107 | 1.245197324 |  | 0.83583114 | 1.06603565 |
|  | 0.938247181 | 0.978614518 |  | 1.214696227 | 1.251713162 |  | 0.8453753 | 1.11879248 |
|  | 0.930002506 | 0.995752786 |  | 1.231765242 | 1.27419605 |  |  |  |
| **Min** | **0.854842318** | **0.937277184** |  | **1.055617383** | **1.245197324** |  | **0.83583114** | **1.06603565** |
| **Q1** | **0.90682091** | **0.967108437** |  | **1.180600821** | **1.267628511** |  | **0.86505822** | **1.10521755** |
| **Median** | **0.932384628** | **0.996309087** |  | **1.203824402** | **1.279160906** |  | **0.91132277** | **1.13341097** |
| **Q3** | **0.959988568** | **1.006007378** |  | **1.224523937** | **1.302042447** |  | **0.93598967** | **1.17499272** |
| **Max** | **1.120783689** | **1.018326948** |  | **1.438343182** | **1.329944629** |  | **1.00756806** | **1.25630243** |
| **Mean** | **0.93901673** | **0.985914337** |  | **1.207450753** | **1.28219603** |  | **0.91048505** | **1.14928476** |
| **Range** | **0.26594137** | **0.081049764** |  | **0.382725798** | **0.084747305** |  | **0.17173692** | **0.19026679** |

**Supplementary table S2. Antibodies and TaqMan probes used in this study.**

| **Marker** | **Host** | **Company** | **Catalog Number** | **Application** |
| --- | --- | --- | --- | --- |
| MYL7 | recombinant human | Miltenyi Biotec | 130-106-142 | Flow Cytometry |
| TNNT2 | recombinant human | Miltenyi Biotec | 130-119-575 | Flow Cytometry |
| Nkx-2.5 | Rabbit | Santa Cruz Biotechnology | sc-14033 | Flow Cytometry |
| Nkx-2.5 | Rabbit | abcam | ab35842 | Flow Cytometry |
| Anti-MLC2v-PE, human, mouse, rat | Rabbit | Miltenyi Biotec | 130-119-680 | Flow Cytometry |
| Anti CD140b-PE, human | recombinant human | Miltenyi Biotec | 130-105-321 | Flow Cytometry |
| Anti CD144 (VE-Cadherin)-FITC, human | recombinant human | Miltenyi Biotec | 130-100-713 | Flow Cytometry |
| FcR Blocking Reagent, human |  | Miltenyi Biotec | 130-059-901 | Flow Cytometry |
|  |  |  |  |  |
| **Marker** | **Host** | **Company** | **Catalog Number** | **Application** |
| ACTA2 | Mouse | abcam | ab7817 | IF |
| CDH5 | Rabbit | abcam | ab33168 | IF |
| GJA1 | Rabbit | abcam | ab11370 | IF |
| MYL7 | Mouse | abcam | ab68086 | IF |
| TNNT2 | Mouse | abcam | ab8295 | IF |
| TNNI3 | Rabbit | abcam | ab47003 | IF |
| Nkx2-5 | Rabbit | abcam | ab35842 | IF |
| ISL1 | Rabbit | abcam | ab20670 | IF |
|  |  |  |  |  |
| **Marker** |  | **Company** | **Catalog Number** | **Application** |
| Human GAPD |  | Thermo Fisher | Hs02786624_g1 | TaqMan Probe |
| Beta ACT (ACTB) |  | Thermo Fisher | Hs01060665_g1 | TaqMan Probe |
| Nanog |  | Thermo Fisher | Hs02387400_g1 | TaqMan Probe |
| POU5F1 (OCT 4) |  | Thermo Fisher | Hs00999634_gH | TaqMan Probe |
| DNMT3B |  | Thermo Fisher | Hs00171876_m1 | TaqMan Probe |
| MESP1 |  | Thermo Fisher | Hs01001283_g1 | TaqMan Probe |
| ISL1 |  | Thermo Fisher | Hs00158126_m1 | TaqMan Probe |
| MIXL1 |  | Thermo Fisher | Hs00430824_g1 | TaqMan Probe |
| NKX2-5 |  | Thermo Fisher | Hs00231763_m1 | TaqMan Probe |
| TMEM88 |  | Thermo Fisher | Hs00396750_g1 | TaqMan Probe |
| ATP2A2 |  | Thermo Fisher | Hs00544877_m1 | TaqMan Probe |
| MYH6 |  | Thermo Fisher | Hs01101425_m1 | TaqMan Probe |
| MYH7 |  | Thermo Fisher | Hs01110632_m1 | TaqMan Probe |
| TNNI1 |  | Thermo Fisher | Hs00913333_ml | TaqMan Probe |
| TNNI3 |  | Thermo Fisher | Hs00165957_m1 | TaqMan Probe |
| TNNT2 (cTnT) |  | Thermo Fisher | Hs00943911_m1 | TaqMan Probe |
| MYL7-(MLC2a) |  | Thermo Fisher | Hs01085598_g1 | TaqMan Probe |
| MYL2 (MLC2v) |  | Thermo Fisher | Hs00166405_m1 | TaqMan Probe |
| HOPX |  | Thermo Fisher | Hs05028646_s1 | TaqMan Probe |
