# Supplementary material for: A method for differentiating human induced pluripotent stem cells toward functional cardiomyocytes in 96-well microplates
Source: Sci Rep. 2020 Oct 28;10:18498. doi: 10.1038/s41598-020-73656-2 (PMC7595118; doi:10.1038/s41598-020-73656-2)
Supplement: Supplementary file 8 — Supplementary file8 [file 41598_2020_73656_MOESM8_ESM.docx]

SUPPLEMENTARY FIGURE LEGENDS AND TABLES

**A method for differentiating human induced pluripotent stem cells toward functional cardiomyocytes in 96-well microplates**

Novin Balafkan, Sepideh Mostafavi, Manja Schubert, Richard Siller, Xiao Liang, Gareth Sullivan, Laurence A. Bindoff

**Supplementary Figure S1. Seeding hPSC in 96well plate at different cell densities.**

The figure shows the morphology of AG05836B-15 cells cultured at different cell density one day after (panels A) and two days (panels B) after seeding in 96 well plates. The A panels show the cells cultured for 24 hrs in E8 supplemented by Y27632, while the B panels show the cells cultured for a further 24 hrs in E8 alone. We tested the following seeding densities 1.2 x 10^4^ (i), 2.4 x 10^4^ (ii), 4.8 x 10^4^ (iii) and 7.2 x 10^4^ cells/cm^2^ (iv). We found that the best cell density for seeding cells to achieve 60-70% confluency prior to the start of differentiation was 2.4 x 10^4^ cells/cm^2^ (equal to 8000 cells per one well of 96 well plate). This cell density gave cells enough time to recover from the Y27632 effects in E8 medium (Aii) and reach to the optimal confluency (Bii). Cells seeded at lower density (1.2 x 10^4^ cells/cm^2^: panels (i)) required more time to reach optimal confluency while seeding cells at higher densities gave rise to very confluent cultures one day after seeding and resulted in poor differentiation efficiency (Panels (iii) and (iv) are representative of 4.8 x 10^4^ cells/cm^2^  and 7.2 x 10^4^ cells/cm^2^ respectively). The effect of confluency on differentiation efficiency is shown in Figure.2.

**Supplementary Figure S2. Characterization of Detroit 551-A-derived cardiomyocytes by gene expression.** Relative quantification of mRNA expression profile of 15 markers, representing different stages of differentiation from hiPSC (D0) to mesodermal layer (D3) following by cardiac progenitor state (D5) and committed cardiomyocyte on D15. Treatment of hiPSC with CHIR99021led to expression of mesodermal markers such as *MESP1* and *MIXL1* on D3. Expression of *ISL1* on D5 following the application of the inhibitor of WNT production-2, IWP2, indicated the presence of cardiac progenitors in the culture. Substantial increase in expression of committed cardiomyocyte markers such as *TBX5*, *TNNT2, MYH6* and *MYL7* revealed the presence of committed cardiomyocytes on D15 of differentiation. Data are presented as the mean of three independent differentiation runs and error bars are the standard deviation of the mean. Changes in expression level are related to the pluripotent stage, D0.

**Supplementary Figure S3.** A) Gene expression study showed a drop of the pluripotency marker *POU5F1* and the appropriate increase in the level of different cardiac lineage markers. Data are collected from experiments using hESC-H9 for cardiac differentiation and presented as mean of 3 independent experiments and error bars are standard deviation of the mean. Changes in expression levels are related to hiPSC, i.e. Day 0. B) Expression of pluripotency marker (POU5F1) and lack of expression of cardiac specific marker (TNNT2) in hiPSC on D0. TNNT2 positive cells were detectable from day 7 where expression of POU5F1 is highly reduced. Images are taken using an epifluorescent microscope (LEITZ DMRBE, Leica). Scale bars, 50μm.

**Supplementary Figure S4**. RT-qPCR analysis of aged cardiomyocyte gene expression. This shows the relative quantification of mRNA expression of *HOPX, MYH7 MYH6* and *NKX2.5.* These results show that cardiomyocytes on D30 express age appropriate markers. This was performed one run of H1 differentiation.

**Supplementary Figure S5.** A) Representative histogram plots showing the fluorescence minus one **(**FMO), isotype and unstained controls of surface markers. The committed cardiomyocytes were gated for CD140b (PE) and CD144 (FITC) and gates were drawn by using FMO together with isotype control to analyze these surface markers. B) Representative histogram plot of fluorescence minus one **(**FMO) control of internal marker TNNT2 (FITC). Graphs were prepared and presented using FlowJo 10.5.0 (FlowJo LLC, OR, USA) software.
